# Cross-neutralization of Omicron BA.1 against BA.2 and BA.3 SARS-CoV-2

**DOI:** 10.1038/s41467-022-30580-5

**Published:** 2022-05-26

**Authors:** Jing Zou, Chaitanya Kurhade, Hongjie Xia, Mingru Liu, Xuping Xie, Ping Ren, Pei-Yong Shi

**Affiliations:** 1 Department of Biochemistry and Molecular Biology, University of Texas Medical Branch, Galveston, TX USA; 2 Department of Pathology, University of Texas Medical Branch, Galveston, TX USA; 3 Sealy Institute for Drug Discovery, University of Texas Medical Branch, Galveston, TX USA; 4 Institute for Human Infection and Immunity, University of Texas Medical Branch, Galveston, TX USA; 5 Institute for Translational Sciences, University of Texas Medical Branch, Galveston, TX USA; 6 Sealy Institute for Vaccine Sciences, University of Texas Medical Branch, Galveston, TX USA

**Keywords:** SARS-CoV-2, Antibodies, Viral infection

## Abstract

The Omicron SARS-CoV-2 has several distinct sublineages, among which sublineage BA.1 is responsible for the initial Omicron surge and is now being replaced by BA.2 worldwide, whereas BA.3 is currently at a low frequency. The ongoing BA.1-to-BA.2 replacement underscores the importance to understand the cross-neutralization among the three Omicron sublineages. Here we test the neutralization of BA.1-infected human sera against BA.2, BA.3, and USA/WA1-2020 (a strain isolated in late January 2020). The BA.1-infected sera neutralize BA.1, BA.2, BA.3, and USA/WA1-2020 SARS-CoV-2s with geometric mean titers (GMTs) of 445, 107, 102, and 16, respectively. Thus, the neutralizing GMTs against heterologous BA.2, BA.3, and USA/WA1-2020 are 4.2-, 4.4-, and 28.4-fold lower than the GMT against homologous BA.1, respectively. These findings have implications in COVID-19 vaccine strategy.

## Abstract (web summary)

It is important to understand the cross-neutralization among distinct SARS-CoV-2 Omicron sublineages. Zou et al. show that sera from Omicron BA.1-infected people are significantly weaker in neutralizing Omicron BA.2, BA.3, and USA-WA1/2020 than neutralizing BA.1.

## Introduction

Since the emergence of severe acute respiratory syndrome coronavirus 2 (SARS-CoV-2) in late 2019, the virus has evolved to increase viral transmission and immune evasion. The World Health Organization (WHO) has so far designated 5 variants of concern (VOC), including Alpha, Beta, Gamma, Delta, and Omicron. At the time of submitting this paper, the newly emerged Omicron variant had 3 distinct sublineages: BA.1, BA.2, and BA.3. BA.1 was first identified in South Africa in November 2021. BA.1 and its derivative BA.1.1 (containing an extra R346K substitution in the spike of BA.1) caused the initial surges of Omicron around the world. Subsequently, the frequency of BA.2 increased steeply, replacing BA.1 in many parts of the world. In the USA, the frequency of BA.2 increased from 0.4% to 54.9% between 22 January 2022 and 24 March 2022 (https://covid.cdc.gov/covid-data-tracker/#variant-proportions). Compared with BA.1, BA.2 did not seem to cause more severe disease^1^, but may increase viral transmissible by ~30%^2^. As of 30 March 2022, the frequency BA.3 remained low in the GISAID database (https://www.gisaid.org/). All three sublineages of Omicron could significantly evade vaccine-elicited neutralization, among which BA.3 exhibited the greatest reduction^3,4^. In addition, Omicron BA.1 could efficiently evade non-Omicron SARS-CoV-2 infection-elicited neutralization^5^. The increased transmissibility and immune evasion of the Omicron variant may be responsible for the replacement of VOC from the previous Delta to the current Omicron. Many unvaccinated individuals were infected by BA.1 during the initial Omicron surge^6^. Therefore, in this work, we examine the cross-neutralization of BA.1 infection against BA.2, BA.3, and other variants. Such laboratory information is essential to guide vaccine strategy and public health policy.

## Results and discussion

**Fig. 1:**
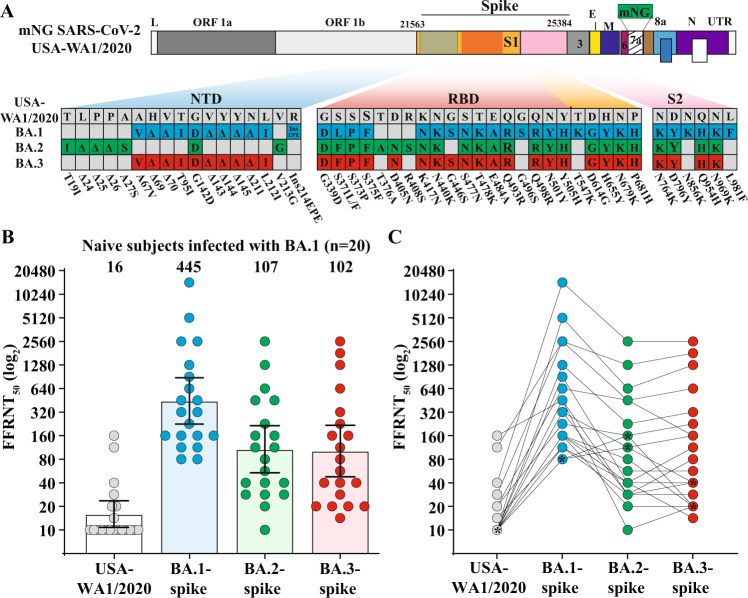
Cross-neutralization of human sera from unvaccinated individuals who were infected with Omicron BA.1 SARS-CoV-2. **A** Omicron BA.1-, BA.2-, and BA.3-spike mNG SARS-CoV-2s. The full-length spike gene from Omicron BA.1, BA.2, or BA.3 was engineered into an mNG USA-WA1/2020 SARS-CoV-2. The mNG gene was engineered at the open-reading-frame-7 of the viral genome. Amino acid mutations, deletions, and insertions (Ins) are indicated for BA.1, BA.2, and BA.3 spikes in reference to the USA-WA1/2020 spike. L leader sequence, ORF open reading frame, NTD N-terminal domain of S1, RBD receptor binding domain of S1, S spike glycoprotein, S1 N-terminal furin cleavage fragment of S, S2 C-terminal furin cleavage fragment of S, E envelope protein, M membrane protein, N nucleoprotein, UTR untranslated region. **B** Scatterplot of neutralization titers. A panel of 20 human sera collected from Omicron BA.1-infected individuals were tested for the 50% fluorescent focus-reduction neutralization titers (FFRNT_50_) against recombinant USA-WA1/2020 (gray circles), Omicron BA.1- (blue circles), BA.2- (green circles), and BA.3-spike (red circels) mNG SARS-CoV-2s. The neutralization titer for each virus was determined in duplicates. The serum information and FFRNT_50_ values are summarized in Table 1. Each data point represents the geometric mean FFRNT_50_ obtained with a serum specimen against the indicated virus. The bar heights and the numbers above indicate geometric mean titers (GMTs). Error bars indicate the 95% confidence interval (CI) of the GMTs. Data are presented as GMT with 95% CI. Statistical analysis was performed using the Wilcoxon matched-pairs signed-rank test. Two-tailed *P* values of the GMT against BA.1-spike and USA-WA1/2020, BA.2-spike, or BA.3 spikes are all <0.0001. **C** FFRNT_50_ values with connected lines for individual sera. Two sera exhibiting slightly higher FFRNT_50_s against BA.2 virus than that against BA.1-spike SARS-CoV-2 are indicated by symbol asterisk (serum ID 1 and 2 in Table 1).

To examine the cross-neutralization among the three Omicron sublineages, we collected 20 human sera from unvaccinated patients who were infected with Omicron BA.1 (Table 1). The genotype of infecting virus was verified for each patient by Sanger sequencing. The sera were collected on day 8 to 62 after positive RT-PCR test. The serum panel was measured for neutralization against four recombinant SARS-CoV-2s (Fig. 1A): USA/WA1-2020 (wild-type) and three chimeric USA/WA1-2020 bearing the full-length spike protein from Omicron BA.1 (GISAID EPI_ISL_6640916), BA.2 (GISAID EPI_ISL_6795834.2), or BA.3 (GISAID EPI_ISL_7605591). The spike proteins of the three Omicron sublineages have distinct amino acid mutations, deletions, and insertions (Fig. 1A). To facilitate neutralization testing, an mNeonGreen (mNG) reporter was engineered into the four viruses, resulting in wild-type, BA.1-, BA.2-, and BA.3-spike mNG SARS-CoV-2s. The construction and characterization of the four mNG SARS-CoV-2s were recently reported^4^. Using an mNG-based fluorescent focus-reduction neutralization test (FFRNT), we determined the neutralizing geometric mean titers (GMTs) of the sera against wild-type, BA.1-, BA.2-, and BA.3-spike mNG SARS-CoV-2s to be 16, 445, 107, and 102, respectively (Fig. 1B). Thus, the neutralizing GMTs against heterologous BA.2-spike, BA.3-spike, and wild-type viruses were 4.2-, 4.4-, and 28.4-fold lower than the GMT against the homologous BA.1-spike virus, respectively (Fig. 1B). Consistently, all sera neutralized BA.1-spike virus at neutralizing titers of ≥80, whereas 13 out of 20 sera did not neutralize the wild-type USA/WA1-2020 (defined as 10 for plot and calculation purposes; Fig. 1B and Table 1). Notably, 2 sera neutralized BA.2-spike virus more efficiently than the BA.1-spike virus (indicated by symbol * in Fig. 1C). Collectively, the results support two conclusions. First, BA.1 infection elicited similar levels of cross-neutralization against BA.2 and BA.3, although at a decreased efficiency that was 4.2- to 4.4-fold lower than that against BA.1. This result is in contrast with the neutralization results from vaccinated sera (collected at 1 month after three doses of Pfizer/BioNTech’s BNT162b2 vaccine) which neutralized BA.1 and BA.2 much more efficiently than BA.3^4^. Second, the neutralization of BA.1-infected sera against USA/WA1-2020 were 6.7- and 6.4-fold lower than that against Omicron BA.2 and BA.3, respectively. The results indicate the antigenic distinctions among different variant spikes, which must be carefully considered when deciding to switch the vaccine sequence to new variants^7^. If future variants are Omicron decedents, it would be conceptually attractive to switch vaccine sequence to an Omicron spike.

**Table 1:** Serum information and FFRNT_50_ values.

Serum ID	Age (years)	Gender (F/M)	Ethnicity	FFRNT_50_^a^				Serum collection time (days post positive RT-PCR test)
				USA-WA1/2020	BA.1-spike virus	BA.2-spike virus	BA.3-spike virus	
1	21–30	M	Hispanic	10^b^	80	160	20	26
2	41–50	F	White	10	80	113	40	33
3	81–90	M	White	14	113	40	14	21
4	11–20	M	Black	10	113	28	28	16
5	31–40	F	Black	10	160	40	40	16
6	21–30	F	Black	10	160	20	20	28
7	1–10	F	Hispanic	10	160	57	57	43
8	61–70	M	Black	10	160	80	20	40
9	81–90	M	White	20	226	160	226	40
10	1–10	F	Hispanic	10	320	28	40	26
11	1–10	F	Hispanic	10	320	113	160	56
12	51–60	F	White	10	453	28	80	35
13	71–80	M	Hispanic	160	453	10	20	17
14	21–30	F	Hispanic	20	640	453	160	62
15	51–60	M	White	10	905	40	113	29
16	71–80	F	NA	10	1280	226	320	8
17	71–80	F	Hispanic	28	2560	453	640	32
18	61–70	M	Hispanic	113	2560	1280	1810	15
19	71–80	F	Hispanic	10	5120	640	1280	16
20	81–90	M	White	40	14,482	2560	2560	13
GMT^c^	–	–	–	16	445	107	102	25
95% CI^d^	–	–	–	11–24	225–881	54–215	48–218	20–32

*NA* not available.

^a^Individual FFRNT_50_ value is the geometric mean of duplicate FFRNT results.

^b^FFRNT_50_ of <20 was treated as 10 for plot purpose and statistical analysis.

^c^Geometric mean neutralizing titers (GMT).

^d^95% confidence interval (95% CI) for the GMT.

There are two limitations of the serum specimens used in this study. First, the sample size of serum specimen was relatively small. Second, all sera were collected on days 8 to 62 after positive RT-PCR test. The immune status of these specimens were heterogenous, with some sera collected at an acute plasma blast stage and other sera collected at a convalescent IgG-dominant phase. Analysis of more specimens collected at later timepoints post-infection will substantiate the current observations.

Emerging evidence supports a vaccine booster strategy to minimize the health risk of the ongoing Omicron infection. First, 2 doses of BNT162b2 vaccine are inefficient to elicit robust neutralization against Omicron variant, whereas 3 doses of BNT162b2 produces robust neutralization against Omicron. Although Omicron-neutralizing activity remains robust for up to 4 months^3^, the durability of such neutralization beyond 4 months after dose 3 remains to be determined. The latter result, together with the real-world vaccine effectiveness, are required to guide the timing of dose 4 vaccine. Second, non-Omicron SARS-CoV-2 infection does not elicit robust neutralization against Omicron variant^5^, suggesting that previously infected individuals should be vaccinated to mitigate the health threat from Omicron. The cross-neutralization of BA.1-infected sera against BA.2 and BA.3 suggests the recent BA.1-infected individuals are likely to be protected against the ongoing BA.2 surge. Third, vaccine-mediated T cell immunity and non-neutralizing antibodies that mediate antibody-dependent cytotoxicity could also confer protection against severe COVID-19. After vaccination or infection, the majority of T cell epitopes are highly preserved against Omicron spikes^8^. In agreement with this notion, 3 doses of BNT162b2 conferred efficacy against Omicron disease, but the protection wanes over time, with overall efficacy remaining high up to 6 months after dose 3^9–13^. The real-world vaccine effectiveness and laboratory studies will guide vaccine booster strategy to achieve optimal breadth and duration of protection.

## Methods

### Ethical statement

All virus work was performed in a biosafety level 3 (BSL-3) laboratory with redundant fans in the biosafety cabinets at The University of Texas Medical Branch at Galveston. All personnel wore powered air purifying respirators (Breathe Easy, 3 M) with Tyvek suits, aprons, booties, and double gloves.

The research protocol regarding the use of human serum specimens was reviewed and approved by the University of Texas Medical Branch (UTMB) Institutional Review Board (IRB number 20-0070). No informed consent was required since these deidentified sera were leftover specimens before being discarded. No diagnoses or treatment was involved either.

### Cells

Vero E6 (ATCC® CRL-1586) were purchased from the American Type Culture Collection (ATCC, Bethesda, MD), and maintained in a high-glucose Dulbecco’s modified Eagle’s medium (DMEM) supplemented with 10% fetal bovine serum (FBS; HyClone Laboratories, South Logan, UT) and 1% penicillin/streptomycin at 37 °C with 5% CO_2_. All culture media and antibiotics were purchased from ThermoFisher Scientific (Waltham, MA). The cell line was tested negative for mycoplasma.

### Recombinant Omicron spike mNG SARS-CoV-2s

The construction and characterization of recombinant Omicron BA.1-, BA.2-, and BA.3-spike mNG SARS-CoV-2s were recently reported^4^. The BA.1, BA.2, and BA.3 spike sequences were derived from GISAID EPI_ISL_6640916, EPI_ISL_6795834.2, and EPI_ISL_7605591, respectively. Passage 1 (P1) virus stocks were produced from infectious cDNA clones of corresponding viruses^14,15^. The P1 viruses were used for neutralization testing throughout the study. The spike gene from each P1 virus was sequenced to ensure no undesired mutations. Equivalent specific infectivities, defined by the genomic RNA-to-FFU (fluorescent focus-forming unit) ratios, were confirmed for individual recombinant P1 virus stocks, as previously reported^4^.

### Serum specimens

The de-identified human sera from unvaccinated patients who were infected by Omicron sublineage BA.1 were heat-inactivated at 56 °C for 30 min before neutralization testing. The genotype of infecting virus was verified by the molecular tests with FDA’s Emergency Use Authorization and Sanger sequencing. The serum information is presented in Table 1.

### Fluorescent focus reduction neutralization test

Neutralization titers of sera were measured by fluorescent focus reduction neutralization test (FFRNT) using the USA-WA1/2020, BA.1-, BA.2-, and BA.3-spike mNG SARS-CoV-2s. The FFRNT protocol was reported previously^5^. Briefly, Vero E6 cells were seeded to 96-well plates at 2.5×10^4^ per well (Greiner Bio-one™). On the following day, heat-inactivated sera were 2-fold serially diluted in culture medium with the first dilution of 1:20 (final dilutions ranging from 1:20 to 1:20,480). The diluted serum was incubated with 100–150 FFUs of indicated mNG SARS-CoV-2s at 37 °C for 1 h. Afterwards, the serum-virus mixtures were loaded onto the pre-seeded Vero E6 cell monolayer in 96-well plates. After 1 h infection, the inoculum was aspirated and overlay medium (100 μl supplemented with 0.8% methylcellulose) was added to each well. After incubating the plates at 37 °C for 16–18 h, raw images of mNG foci were acquired using Cytation^TM^ 7 (BioTek) with Gene5 software. The foci in each well were counted and normalized to the no-serum-treated controls to calculate infection rates. The FFRNT_50_ value was defined as the minimal serum dilution that suppressed >50% of fluorescent foci. The neutralization titer of each serum was determined in duplicates, and the geometric mean was presented. Figures were initially plotted using GraphPad Prism 9.0 software, and assembled in Adobe Illustrator. FFRNT_50_ of <20 was treated as 10 for plot purpose and statistical analysis; in this way, we can differentiate between sera with FFRNT_50_ of 20 and sera with no neutralization activity at 1:20 dilution (treated as FFRNT_50_ of 10). Table 1 summarizes the FFRNT_50_ results.

### Statistics

The nonparametric Wilcoxon matched-pairs signed-rank test was used to analyze the statistical significance in Fig. 1B.

### Reporting summary

Further information on research design is available in the Nature Research Reporting Summary linked to this article.

## Supplementary information

Reporting Summary

## Data Availability

The raw data that support the findings of this study are shown in the Source data file. Source data are provided with this paper.

## Source data

Source Data
