# Supplementary material for: Cross-neutralization of Omicron BA.1 against BA.2 and BA.3 SARS-CoV-2
Source: Nat Commun. 2022 May 26;13:2956. doi: 10.1038/s41467-022-30580-5 (PMC9135681; doi:10.1038/s41467-022-30580-5)
Supplement: Supplementary file 1 — Reporting Summary [file 41467_2022_30580_MOESM1_ESM.pdf]

nature portfolio

Corresponding author(s): Pei-Yong Shi
Last updated by author(s): 5/4/2022

Reporting Summary

Nature Portfolio wishes to improve the reproducibility of the work that we publish. This form provides structure for consistency and transparency in reporting. For further information on Nature Portfolio policies, see our Editorial Policies and the Editorial Policy Checklist.

Statistics

For all statistical analyses, confirm that the following items are present in the figure legend, table legend, main text, or Methods section.

n/a	Confirmed	
☐	☒	The exact sample size (*n*) for each experimental group/condition, given as a discrete number and unit of measurement
☐	☒	A statement on whether measurements were taken from distinct samples or whether the same sample was measured repeatedly
☐	☒	The statistical test(s) used AND whether they are one- or two-sided<br>*Only common tests should be described solely by name; describe more complex techniques in the Methods section.*
☒	☐	A description of all covariates tested
☒	☐	A description of any assumptions or corrections, such as tests of normality and adjustment for multiple comparisons
☐	☒	A full description of the statistical parameters including central tendency (e.g. means) or other basic estimates (e.g. regression coefficient) AND variation (e.g. standard deviation) or associated estimates of uncertainty (e.g. confidence intervals)
☐	☒	For null hypothesis testing, the test statistic (e.g. *F*, *t*, *r*) with confidence intervals, effect sizes, degrees of freedom and *P* value noted<br>*Give P values as exact values whenever suitable.*
☒	☐	For Bayesian analysis, information on the choice of priors and Markov chain Monte Carlo settings
☒	☐	For hierarchical and complex designs, identification of the appropriate level for tests and full reporting of outcomes
☒	☐	Estimates of effect sizes (e.g. Cohen's *d*, Pearson's *r*), indicating how they were calculated

Our web collection on statistics for biologists contains articles on many of the points above.

Software and code

Policy information about availability of computer code

Data collection	Cytation7 with Gene 5 software
Data analysis	GraphPad Prism 9.0

For manuscripts utilizing custom algorithms or software that are central to the research but not yet described in published literature, software must be made available to editors and reviewers. We strongly encourage code deposition in a community repository (e.g. GitHub). See the Nature Portfolio guidelines for submitting code & software for further information.

Data

Policy information about availability of data

All manuscripts must include a data availability statement. This statement should provide the following information, where applicable:

- Accession codes, unique identifiers, or web links for publicly available datasets
- A description of any restrictions on data availability
- For clinical datasets or third party data, please ensure that the statement adheres to our policy

The raw data that support the findings of this study are shown in the Source data file.

Field-specific reporting

Please select the one below that is the best fit for your research. If you are not sure, read the appropriate sections before making your selection.

☒ Life sciences ☐ Behavioural & social sciences ☐ Ecological, evolutionary & environmental sciences

For a reference copy of the document with all sections, see nature.com/documents/nr-reporting-summary-flat.pdf

Life sciences study design

All studies must disclose on these points even when the disclosure is negative.

Sample size	No sample size calculation was performed. Based on the availability, 20 samples SARS-CoV-2 BA.1-infected samples were collected.
Data exclusions	No data was excluded in the study.
Replication	Each human serum sample was analyzed in duplication. The averaged results from the duplication were reported in this study. All attempts at replication were successful.
Randomization	This is not relevant to the study. All samples available were analyzed for the neutralizing activities against WT SARS-CoV-2 and variants in the same experimental settings.
Blinding	Patient information was blinded in the study. The investigators were blinded to sample identity during data collection and/or analysis.

Reporting for specific materials, systems and methods

We require information from authors about some types of materials, experimental systems and methods used in many studies. Here, indicate whether each material, system or method listed is relevant to your study. If you are not sure if a list item applies to your research, read the appropriate section before selecting a response.

Materials & experimental systems

n/a	Involved in the study	
☒	☐	Antibodies
☐	☒	Eukaryotic cell lines
☒	☐	Palaeontology and archaeology
☒	☐	Animals and other organisms
☐	☒	Human research participants
☒	☐	Clinical data
☒	☐	Dual use research of concern

Methods

n/a	Involved in the study	
☒	☐	ChIP-seq
☒	☐	Flow cytometry
☒	☐	MRI-based neuroimaging

Eukaryotic cell lines

Policy information about cell lines

Cell line source(s)	Vero E6 cells (ATCC® CRL-1586) were obtained from ATCC
Authentication	Cells have been authenticated by ATCC using morphologies and other groups using STR profiling (reference: Almeida JL, Hill CR, Cole KD. Authentication of African green monkey cell lines using human short tandem repeat markers. BMC Biotechnol. 2011;11:102. Published 2011 Nov 7. doi:10.1186/1472-6750-11-102).
Mycoplasma contamination	All cell lines were tested negative for mycoplasma.
Commonly misidentified lines (See ICLAC register)	None.

Human research participants

Policy information about studies involving human research participants

Population characteristics	Samples were collected based on availability. The population characteristics (age, gender and genotypic information) were reported in this study.
Recruitment	No patients were recruited in this study. No informed consent was required, becasue these deidentified sera were leftover specimens before being discarded. No diagnoses or treatment was involved either.

Ethics oversight	The research protocol regarding the use of human serum specimens was reviewed and approved by the University of Texas Medical Branch (UTMB) Institutional Review Board (IRB#: 20-0070).

Note that full information on the approval of the study protocol must also be provided in the manuscript.
